# Treament of Hæmoptysis by Ergot of Rye

**Published:** 1869-04

**Authors:** Horace Dobell

**Affiliations:** Senior Physician to the Royal Hospital for Diseases of the Chest, &c.


Treatment of Hæmoptysis by Ergot of Rye.—By Horace Dobell, M. D., Senior Physician to the Royal Hospital for Diseases of the Chest, &c., in *British Medical Journal*, June 27.

In spite of the fashionable outcry against complicated prescriptions, Dr. Dobell ventures to give the following as the most efficacious, and, as it seems to him, the most rational, combination of remedies for a case of profuse tubercular pulmonary hemorrhage:—

R.—Ext. ergotæ liq. drachm ij (to contract the vessels); tincturæ digitalis. drachm ij (to steady his heart); acidi gallici, drachm j (to clot the blood); magn. sulphatus, drachm vj (to relieve congestion); acidi sulphurici diluti, drachm j (to assist the rest); infusi rosæ acidi, ad oz.viij (to make a mixture). A sixth part every three hours till hemorrhage is arrested.

In any given case, either of the ingredients may be omitted, if the symptoms indicate that it is not required, or that it has already done its duty.

